# Bibliography

**Published:** 1904-02-15

**Authors:** 


### BIBLIOGRAPHY.

Another volume of the "*Medical Epitome Series,*" published by Lea Brothers & Co., has just been issued. Like its predecessors, it gives a clear and tolerably comprehensible statement of its subject. This one is on the subjects of Organic and Physiological Chemistry. Both of these subjects are so vast and difficult of statement even in the larger treatises, that it should not be expected that much could be given in a small work of two hundred pages, yet the author has condensed so well that every phase of the two subjects has received a fair, though in some cases too brief, statement of the present knowledge of these subjects. As a student reminder and to point out the more important facts this little book has a valuable function. Dr. Alexius McGlannan, of Baltimore College of Physicians and Surgeons, did the editorial work in a very thorough manner.

---

THE fourth edition of Warren's Quiz Compend on Dental Pathology and Dental Medicine has just come from the press of P. Blackistone & Co. As a brief statement of fundamentals it is very good as well as accurate. It can not be expected in so small a work that a very comprehensive statement of the anatomical and pathological facts would be attempted, or that a full statement of the therapeutic use and influence of all drugs indicated in dental practice should be made. The author has confined himself to giving a brief mention only of the different classes of drugs. As is to be expected one looks in vain for a satisfactory and comprehensive statement of dental pathology and treatment. But the work is deserving and serves an excellent purpose as a students' reminder.

